# Endoscopic Self-Expandable Metallic Stent Insertion without Fluoroscopic Guidance Is Feasible and Safe for Acute Colonic Obstruction Caused by Colorectal Cancer

**DOI:** 10.1155/2020/6810164

**Published:** 2020-01-10

**Authors:** Yadong Feng, Qian Yu, Ming Li, Wei Xu, Ye Zhu, Yang Liu, Ruihua Shi

**Affiliations:** Department of Gastroenterology, Zhongda Hospital, School of Medicine, Southeast University, 87 Dingjiaqiao Road, Nanjing, China 210009

## Abstract

**Aims:**

Endoscopic self-expandable metallic stent (SEMS) insertion for acute colonic obstruction caused by colorectal cancer (CRC) is always performed under fluoroscopic guidance. This study evaluated the feasibility and safety of an endoscopic stenting procedure without fluoroscopic guidance.

**Methods:**

A total of 36 patients with an acute colonic obstruction caused by CRC underwent endoscopic SEMS insertion using a colonoscope without fluoroscopic guidance, followed by analyses of the technical and clinical success and short-term complications.

**Results:**

Total technical success rate and clinical success rate were 91.7% and 86.1%, respectively. The mean procedure time was 21.2 ± 10.3 minutes. There was no stent dislodgement. One case of hematochezia and two cases of tenesmus occurred in patients with left-sided complete obstructions. No other short-term complications occurred. Procedure time, technical success, and clinical success rate were 16.3 ± 9.4 minutes, 93.1%, and 89.6% for left-sided obstructions, respectively, and were 26.8 ± 10.7 minutes, 85.7%, and 71.4% for right-sided obstructions, respectively. For complete obstructions, procedure time, technical success, and clinical success rate were 22.5 ± 8.9 minutes, 90%, and 83.3%, respectively. In the incomplete cases, procedure time, technical success, and clinical success were 13.5 ± 6.7 minutes, 100%, and 100%, respectively. Technical success, clinical success, and short-term complications were not differed between lesion locations and degrees.

**Conclusions:**

This simple technique is feasible and safe for palliation of acute colonic obstruction caused by CRC.

## 1. Introduction

Acute colonic obstruction occurs in approximately 7%–29% of patients with colorectal cancer (CRC) [1, 2]. Although not commonly seen, acute colonic obstruction is a worldwide emergency for gastroenterologists and surgeons. Emergency surgery, even including a diverting stoma, is associated with a high frequency of complications and mortality [2, 3]. Endoscopic insertion of self-expanding metallic stents (SEMS) is an established treatment for CRC-induced acute colonic obstruction [4].

The endoscopic colonic stenting is usually performed in the endoscopic unit by using a combined endoscopic and fluoroscopic procedure [4–6]. This hybrid endoscopic/radiological procedure is with the advantage of real-time endoscopic and fluoroscopic guidance; however, the cooperation between the endoscopists and radiologists is needed. If fluoroscopy was in a full schedule, emergency colonic stenting for patients with an acute colonic obstruction in a combined endoscopic and fluoroscopic approach may be delayed; even some patients may be treated with emergency laparotomy or open surgery [6]. If nonradiation stenting insertion was technically feasible, patients with a CRC-induced acute colonic obstruction will get more benefits from a timely stenting. However, nonradiation endoscopic stenting without fluoroscopic guidance for an acute colonic obstruction is not available currently.

Clinically, an abdominal computed tomography (CT) scan is usually performed before SEMS insertion. Some radiological features of acute colonic obstruction, such as colon dilation proximal to the obstruction, location of obstructive lesions, sizes of tumors, and length of strictures, can be revealed by a CT scan. Since endoscopic stenting is an image-guiding procedure, characteristics of preprocedural imaging make it possible for SEMS insertion for CRC-induced acute colonic obstruction with a therapeutic colonoscopy only.

In this study, we conducted emergency SEMS insertions for acute colonic obstruction caused by CRC using endoscopic approach without fluoroscopy guidance. All SEMS insertions were performed according to the prestenting image, by using a colonoscope only and without the guidance of fluoroscopy. The objective of this study was to evaluate the efficacy and feasibility of this technique using SEMS insertion.

## 2. Patients and Methods

### 2.1. Clinical Data

From January 2016 to December 2018, 36 patients with CRC obstruction underwent endoscopic SEMS insertion in our center. Abdominal contrast CT scan was examined to confirm that CRC caused colonic obstruction. The location and size of tumors and length of strictures were evaluated. Sizes of SEMS were determined by the images from the CT scan. Informed consent was obtained from all patients. Characteristics of the clinical data are listed in Table 1.

### 2.2. SEMS Insertion

Informed consent was signed before SEMS insertion in all patients. Cleansing enemas were given before SEMS insertion. Patients underwent deep sedation by using propofol intravenously. All SEMS insertion procedures were performed by experienced endoscopists. A 3.7 mm single channel colonoscope (CF-260H; Olympus Medical Systems, Tokyo, Japan) was used. A Niti-S uncovered colorectal stent with a diameter of 22–25 mm and a length of 100 mm from MicroTech (Nanjing, China) or Cook Medical Evolution (Bloomington, IN, USA) was used according to the endoscopist's preference.

**Figure 1:**
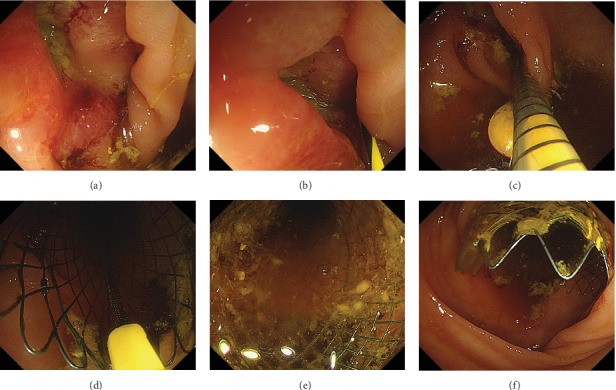
The procedures of one SEMS insertion. (a) Endoscopic view of a complete colonic malignant obstruction. (b) A hydrophilic 0.035-inch biliary guidewire was used to traverse the stricture. (c) The delivery system was advanced over the guidewire and guided into the site of obstruction. (d) Endoscopic view of stent release. (e) Endoscopic view of SEMS expansion after release. (f) The distal end of SEMS was about 2 cm beyond the edge of the tumor after stent deployment.

The whole stenting procedure was monitored under endoscopic guidance only. A through-the-scope technique was used for all stent insertions. Briefly, when the colonoscope reached the obstruction, a sphincterotome (MicroTech, Nanjing, China) for endoscopic retrograde cholangiopancreatography (ERCP) with a 0.035-inch guidewire (MicroTech, Nanjing, China) or Jagwire (Boston Scientific, Natick, MA, USA) was introduced. The guidewire was inserted to traverse the stricture. The guidewire was inserted for approximately 40 cm, then pulled backward at approximately 10 cm, followed by an additional insertion for approximately 20 cm. Successful guidewire insertion was judged by a smooth passage of the guidewire without any resistance. The sphincterotome was then inserted along the guidewire to confirm the passage of the obstruction. The delivery system was advanced over the guidewire and was guided into the site of obstruction until the distal end of the stent was approximately 2 cm at the edge of the tumor. In the stent deployment procedure, the distal end of the stent was kept 2 cm beyond the distal edge of the lesion, while the outer sheath was retracted. The whole procedure was monitored by the colonoscopic guidance only. The procedures involving a SEMS insertion are shown in Figure 1.

The whole manipulation time of SEMS implantation was calculated. Technical success was defined as the deployment of the stent across the entire length of the stricture. Clinical success was defined by the resolution of symptom relief of the obstruction within 24 hours. Possible short-term complications included hematochezia, perforation, stent migration, and tenesmus.

### 2.3. Statistics

Continuous variables are expressed as the mean ± standard deviation (SD). The chi-square test and one-way analysis of variance (ANOVA) were performed using SPSS statistical software for Windows, version 20.0 (SPSS Inc., Chicago, IL, USA). A *p* value < 0.05 was considered to be statistically significant.

## 3. Results

Baseline demographic characteristics are summarized in Table 1. Thirty-one cases were primary CRC, and five cases were recurrent tumors. All patients had occlusive symptoms. Twenty-nine patients had an obstruction at the left-sided colon, and the remaining seven patients had a right-sided colon obstruction. Thirty cases were complete obstruction. According to CT scans, the length of an obstruction was 6.2 ± 1.36 cm.

The outcomes of all SEMS insertions are listed in Table 2. There were three cases of technical failure due to failure of guidewire passage through a complete obstruction. Two were left-sided CRC and one was a right-sided obstruction. These patients transferred to endoscopy and fluoroscopy-combined stent insertion, but technical success was not achieved. They subsequently underwent a laparotomy for a diverting stoma. The mean time of the remaining 33 cases of stent implantations was 21.2 ± 10.3 minutes. There was no stent dislodgement. Total technical success rate and clinical success rate were 91.7% and 86.1%, respectively. After stent insertion, one patient suffered from hematochezia and two patients suffered from tenesmus, and all patients obtained symptom relief after conservative therapy. No other short-term complications occurred.

Outcomes from subgroups were analyzed, and the results are listed in Table 3. Procedure time, technical success rate, and clinical success rate were 16.3 ± 9.4 minutes, 93.1%, and 89.6%, respectively, for left-sided obstructions and were 26.8 ± 10.7 minutes, 85.7%, and 71.4%, respectively, for right-sided obstructions. For complete obstructions, procedure time, technical success rate, and clinical success rate were 22.5 ± 8.9 minutes, 90%, and 83.3%, respectively. In the incomplete cases, procedure time, technical success rate, and clinical success rate were 13.5 ± 6.7 minutes, 100%, and 100%, respectively. Hematochezia and tenesmus occurred in left-sided CRC patients, but no immediate complication occurred in right-sided CRC patients. All stent insertion-related complications occurred in complete obstruction cases. The mean procedure time was shorter in the left-sided obstructions than that in the right-sided lesions (*p* = 0.001). There was no statistical difference of technical success, clinical success, and short-term complications between different locations and degrees.

## 4. Discussion

Endoscopic stenting for malignant colonic obstruction was firstly reported in the 1990s [7] and has been widely used because of its low invasiveness for intestinal decompression. In clinical practice, SEMS placement is convenient in those incomplete obstructive cases. However, most complete obstructions in CRC are complex and SEMS placement remains difficult and challenging. From the standpoint of safety and efficiency, stenting in CRC is usually performed by using a combined colonoscopic and fluoroscopic approach [4–8], and this hybrid procedure is widely accepted. Characterized by cannulation of strictures, the procedures of endoscopic stenting for colonic obstruction are similar with those of ERCP. Although traditional ERCP is a hybrid image-guided procedure, nonradiation ERCP has been reported in recent years [9], especially in very severe cases and in patients with pregnancy. Theoretically, colonic stenting can be performed by using similar techniques of nonradiation biliary cannulation. Because fluoroscopy in our center was always in a full schedule, we performed the SEMS insertion under endoscopic guidance as the first-line therapy. In the present study, we reported our experience of the endoscopy-guided only method for SEMS insertion.

Since gastrointestinal endoscopy is an image-guided procedure, the therapeutic strategy can be made according to preprocedure images. For example, a CT scan before stent insertion has been emphasized [6, 10], by providing some radiological evidence. In our practice, determinations are made by reviewing images of CT scans. According to the images of the CT scans, we found the mean length of strictures was not beyond 8 cm and cannulation through a stricture with a hydrophilic biliary guidewire was feasible [11]. In difficult cases, such as complete obstructions, a biliary sphincterotome-aided cannulation can facilitate a successful passage of a guidewire through a stricture [11, 12]. The use of a sphincterotome, which can provide an appropriate angle for the guidewire to pass through the obstructing lesion, may increase the success rate of cannulation. We performed cannulation of a stricture with the sphincterotome-aided technique in most cases, and cannulations were achieved with a high success rate. In previous studies, fluoroscopy was used to confirm the successful passage of a guidewire through the obstruction [13, 14]. However, if a guidewire is transversed through a stricture, the insertion of a guidewire will be smooth and without resistance, and the judgement of a successful cannulation can be made by experienced endoscopists. This successful colonic cannulation is similar to the nonradiation biliary cannulation [15, 16]. The second advantage of the endoscope and fluoroscopy-combined technique is that the length of a stricture can be measured using the fluoroscopic monitor [8, 13, 14]. Based on CT scans, strictures were no longer than 10 cm, so commercially available stents 100 mm in length could be used for palliation of colonic obstructions [8]. In our practice, the through-the-scope technique was used for SEMS insertion and stent deployment. By keeping the distal end immobilized, the stent was placed in the correct location with both ends beyond the lesion after deployment. This approach can be performed under endoscopic guidance and allows direct visualization in order to ensure that the stent is properly deployed in the correct location [8]. Compared with a previous study [17], our study had a shorter procedure time, which was the result of an abbreviated procedure without fluoroscopy.

The success rate of SEMS stenting varies in different centers. According to a previous report, Yoshida et al. [17] achieved a 100% success rate and a 97% clinical success rate. However, in another study [5], the technical success rate was approximately 73%. The results of our technical and clinical success rates were similar with those of some previous studies [18–20]. Although stenting for right-sided malignant colonic obstruction has a success rate of approximately 95% [21], it is more technically difficult and challenging than stenting for left-sided lesions. According to previous studies [10, 14, 18], stenting for the right-sided obstructions was with a lower success rate than that in the left-sided lesions. In the present study, although the success rate in right-sided obstructions was lower than that in the left-sided lesions, it was not statistically different. This may be partly due to a limited number of the right-sided cases. In our study, no serious immediate complication occurred. This was mainly because our group was experienced with endoscopic stenting techniques and familiar with the stent deployment systems [22]. Additionally, in our practice, a SEMS at 22 mm in diameter was more frequently used, and a smaller SEMS may be correlated with less adverse events [8].

This study had some limitations. This was a respective and descriptive study. Because acute CRC-caused obstruction is not frequently encountered except for emergency cases in clinics, we recommended endoscopic stenting as the first-line therapy for palliation. This study evaluated the technique of stent implantation, and only technical success, clinical success, and short-term complications were analyzed. In this case series, complications were more frequent in patients with a left-sided colonic obstruction; this may be related with fewer right-sided CRC cases. Additionally, due to the fact that right-sided obstruction cases are rare, the efficacy should be further investigated using more patients.

In conclusion, the results of the present study have shown that SEMS insertion with endoscopic guidance only is efficient and safe, even in complete colonic obstructions. This nonradiation colonic stenting is technically feasible, especially for endoscopists familiar with hepatobiliary and pancreatic interventions.

## Figures and Tables

**Table 1:** Characteristics of patients.

Total patients	36
Gender	
Male (%)	23 (63.9)
Female (%)	13 (36.1)
Age (years)	72.6 ± 10.5 (47-89)
Obstruction location	
Left-sided colon (%)	29 (80.6)
Right-sided colon (%)	7 (19.4)
Complete obstruction (%)	30 (83.3)
Stricture length (cm)	6.2 ± 1.36 (1-8)

**Table 2:** Outcomes of SEMS insertion.

Total technical success, no. (%)	33 (91.7)
Total clinical success, no. (%)	31 (86.1)
Procedure time (min)	21.2 ± 10.3 (12-56)
Short-term complications	
Hematochezia	1
Tenesmus	2
Stent dislodgement	0
Perforation	0
Stent migration	0

**Table 3:** Analyses of SEMS insertion stratified by CRC location and obstruction degree.

	Location		*p* value	Degree		*p* value
	Left-sided (*n* = 29)	Right-sided (*n* = 7)		Complete (*n* = 30)	Incomplete (*n* = 6)	
Technical success, no. (%)	27 (93.1)	6 (85.7)	0.526	27 (90)	6 (100)	0.418
Clinical success, no. (%)	26 (89.6)	5 (71.4)	0.211	25 (83.3)	6 (100)	0.281
Procedure time (min)	16.3 ± 9.4 (12-25)	26.8 ± 10.7 (18-56)	0.00	22.5 ± 8.9 (16-56)	13.5 ± 6.7 (12-25)	0.179
Short-term complications, no.	3	0	0.674	3	0	0.396
Hematochezia, no. (%)	1 (3.45)	0		1 (3.33)	0	
Tenesmus, no. (%)	2 (6.90)	0		2 (6.67)	0	

## Data Availability

The data used to support the findings of this study are included within the article.
